# Implementing a national acute rheumatic fever case-finding program and registry in Nepal: protocol for a multi-site implementation study

**DOI:** 10.3389/fcvm.2026.1823957

**Published:** 2026-05-07

**Authors:** Sheila Klassen, Sandeepa Karki, Alma Adler, Prakash Raj Regmi, Kunjang Sherpa, Ganesh Shetty, Prashant Khadka, Urmila Shakya, Samir Shakya, Neil Gupta, Shela Sridhar, Gene Bukhman, Robert Levine, Bhagawan Koirala

**Affiliations:** 1 Center for Integration Science, Division of Global Health Equity, Brigham and Women’s Hospital, Boston, MA, United States; 2 Kathmandu Institute of Child Health, Kathmandu, Nepal; 3 Department of Global Health and Social Medicine, Harvard Medical School, Boston, MA, United States; 4 Nepal Heart Foundation, Kathmandu, Nepal; 5 National Academy of Medical Sciences, Bir Hospital, Kathmandu, Nepal; 6 Manmohan Cardiothoracic Vascular and Transplant Center, Kathmandu, Nepal; 7 Cardiac Ultrasound Laboratory, Massachusetts General Hospital, Boston, MA, United States

**Keywords:** acute rheumatic fever, case finding, implementation study, Nepal, rheumatic heart disease

## Abstract

**Background:**

Rheumatic heart disease (RHD) is a leading cause of preventable cardiovascular morbidity and mortality among children and young adults in low- and lower-middle-income countries. Acute rheumatic fever (ARF), the immunologic precursor to RHD, is frequently missed in endemic settings, resulting in delayed treatment and progressive valvular disease. Nepal bears a high burden of RHD but lacks a coordinated system for early ARF identification, standardized echocardiographic evaluation, and longitudinal follow-up.

**Methods:**

This protocol describes implementation of a standardized ARF case-finding program and integrated clinical and echocardiographic registry across 11 district and referral hospitals in Nepal. The program is delivered through the PRIMA Network in partnership with the Kathmandu Institute of Child Health (KIOCH), embedded within existing PEN-Plus chronic disease infrastructure at district-level facilities. Case-finding is integrated into routine care using the 2015 modified Jones criteria, standardized echocardiography protocols, centralized training, and REDCap registry-based follow-up. Implementation outcomes are evaluated using Proctor's taxonomy, focusing on acceptability, feasibility, and adoption of the program within routine care, rather than on clinical detection rates or RHD outcomes. Contextual determinants are examined using the Consolidated Framework for Implementation Research (CFIR). Care-process indicators including diagnostic patterns and follow-up completion are summarized descriptively over the first 12 months of program delivery.

**Discussion:**

This protocol describes a pragmatic approach to embedding ARF case-finding within routine district-level care in Nepal. Findings will inform future evaluations of program performance, scale-up, and sustainability, and may serve as a replicable model for strengthening ARF detection in other RHD-endemic low-resource settings.

## 1 Introduction

Rheumatic heart disease (RHD) remains one of the leading causes of preventable cardiovascular morbidity and mortality among children and young adults in low- and lower-middle-income countries (LMICs), including in the South Asia region (1). Untreated Group A streptococcal (GAS) pharyngitis leads to acute rheumatic fever (ARF), an immunologic response which eventually causes the cardiac valve damage characterizing RHD. Within South Asia, Nepal is recognized as an endemic country for RHD, reflecting ongoing transmission of GAS infections and persistent gaps in early detection and prevention.

Nepal is a mountainous country bordered by the Himalayas with the largest rural proportion in the region of South Asia (2). Rural households in hill and mountain districts often face difficult terrain, monsoon, landslides, and floods, that impede timely access to health facilities, particularly for non-emergency conditions such as sore throat. Prevalence of RHD in Nepal is high and increases with age, with an estimated 5.5 per 1,000 children 5 years of age with borderline or definite RHD and 16.0 per 1,000 in children 15 years of age (3) according to school echocardiographic screening studies. Regional variation within Nepal has been found, with higher prevalence of RHD in the Southern region compared to other regions (4). An estimated 20.1% of Nepal's population, approximately six million people, meet criteria for multidimensional poverty (5). The highest levels of poverty are in Southern Nepal, corresponding to the region with the highest RHD prevalence (4).

There have been important efforts by the Nepal Heart Foundation to establish a nationwide RHD registry (4), however most children in Nepal continue to be diagnosed late—after the development of clinically important heart disease. These gaps persist in part because of the low awareness of ARF among clinicians and the community, the lack of standardized echocardiographic assessment at the district level, and the absence of longitudinal clinical registries that track referrals between levels of the health system and follow-up. This program builds on the joint structure of decentralized rural care via PEN-Plus and ARF case-finding for biomarker discovery as part of PRIMA and is made possible by efforts from both Networks. Joint PEN-Plus and PRIMA efforts place an emphasis on ARF case-finding, early detection of ARF and RHD, improved access to care for this population, and high-quality follow-up in rural hospitals.

This implementation protocol, guided by the TIDieR (6) and StaRI (7) reporting frameworks, describes the implementation of an ARF case-finding program and integrated RHD clinical and echocardiographic registry in Nepal, with a focus on evaluating whether this program can be feasibly and acceptably embedded within routine health system care.

### 1.1 Objectives

The primary objective of the study described in this protocol is to assess the acceptability, feasibility and facility-level adoption of the described ARF case-finding and registry program embedded within routine district and referral hospital care across diverse geographic settings in Nepal. This study does not evaluate the clinical effectiveness of ARF case-finding or its impact on RHD outcomes, which are beyond the scope of this early implementation evaluation.

Secondary objectives are: to measure fidelity to standard operating procedures (SOPs), defined as adherence to ARF diagnostic criteria, echocardiographic protocols, and registry documentation requirements; to describe the penetration of ARF case-finding within participating facility catchment areas; to evaluate short-term sustainability, defined as continued program operation and sustained workflow adherence at 12 months; to identify contextual factors associated with variation in adoption and fidelity across KIOCH and referral facilities using selected Consolidated Framework for Implementation Research (CFIR) domains.

## 2 Methods and analysis

### 2.1 Study design

This is a protocol for a prospective, pragmatic, multi-site implementation study conducted across six district and five referral hospitals in Nepal. This observational study evaluates early implementation of a standardized ARF case-finding and registry program embedded within routine inpatient and outpatient care. The primary unit of analysis is the participating facility, reflecting integration of the standardized workflow across Nepal's diverse geographic and socioeconomic health system contexts. Implementation outcomes will be evaluated over the first 12 months of program delivery after a two-month run-in period (April 2026-April 2027) using a mixed-methods, registry-based approach. The study is conducted within the broader PRIMA research platform but focuses specifically on implementation of the ARF case-finding and registry workflow.

Recruitment activities began in February 2026 and will continue on a rolling basis across participating facilities.

### 2.2 Conceptual framework

This study is guided by an implementation science framework based on Proctor's taxonomy of implementation outcomes (8), which is described in Supplementary Table S1. At this early stage of program delivery, we are evaluating the acceptability, adoption, feasibility, fidelity, penetration, and short-term sustainability of a standardized ARF case-finding and registry program embedded within routine clinical care.

Within this framework, acceptability refers to user, provider and other stakeholders perceived perception of the appropriateness of the program, adoption refers to initiation of the standardized workflow across participating facilities, feasibility reflects the extent to which the workflow can be successfully carried out within existing clinical systems, fidelity captures adherence to diagnostic and registry SOPs, and short-term sustainability assesses continued operation at 12 months. Penetration and exploratory care-process indicators are examined to contextualize early implementation performance.

**Figure 1:**
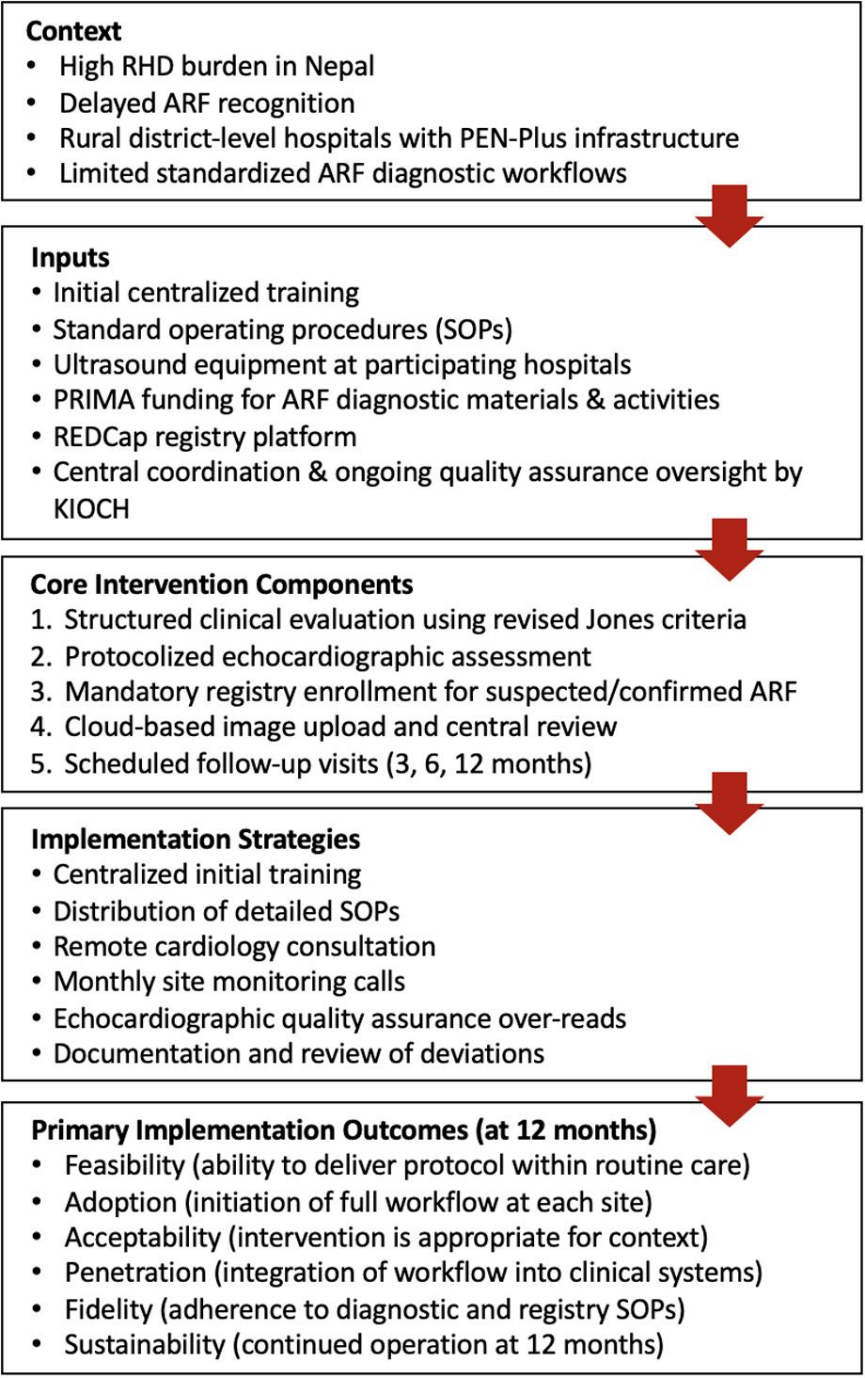
Conceptual model for the ARF case-finding and registry implementation study in Nepal. The model depicts how program inputs and implementation strategies are expected to produce measurable implementation outcomes, which are evaluated during the first 12 months of program delivery.

The conceptual model underlying the program is described in Figure 1. The model depicts contextual factors, program inputs, core intervention components, and implementation strategies leading to evaluation of adoption, feasibility, fidelity, and short-term maintenance. Long-term clinical effectiveness, including impact on ARF incidence or RHD progression, is beyond the scope of this initial implementation evaluation.

### 2.3 Study setting and context

KIOCH is a not-for-profit organization established in 2017 to develop a hub-and-spoke network of multispecialty pediatric facilities in Nepal. KIOCH's mission is to provide timely, accessible, high-quality health care to all Nepali children irrespective of their economic status, with an initial focus on cardiology and cardiothoracic surgery, oncology, mental health, emergency, and critical care. This network, which is still in development, includes a central hub hospital in Budhanilkantha Municipality, Kathmandu, and satellite units in each province, with a strong emphasis on community outreach, school health programs, and district health system strengthening. The Preventing Rheumatic Injury biomarker Alliance (PRIMA) Network has partnered with KIOCH to leverage this emerging infrastructure for the implementation of their biomarker study across KIOCH facilities.

### 2.4 Integrating ARF case-finding into Nepal's health system

PRIMA is a multi-country research network focused on improving early detection of ARF through harmonized clinical phenotyping and biomarker discovery. To support this goal, participating countries implement standardized ARF case identification, clinical characterization, registry infrastructure, and longitudinal follow-up protocols to ensure consistent phenotyping across recruiting facilities.

Many of the participating district-level hospitals in this study are implementing the WHO-endorsed Package of Essential Noncommunicable Disease Interventions–Plus (PEN-Plus) strategy (9), which decentralizes care for severe chronic noncommunicable disease to first-level referral hospitals in low-resource settings, primarily in Sub-Saharan Africa and South Asia (10, 11). The decentralization approach that PEN-Plus uses centers on task-sharing, standardized treatment protocols, and longitudinal follow-up for complex conditions (such as RHD) at district-level hospitals that are more geographically accessible to rural populations than tertiary care centers in urban areas (12), allowing ARF and RHD services to be embedded into routine care.

**Figure 2:**
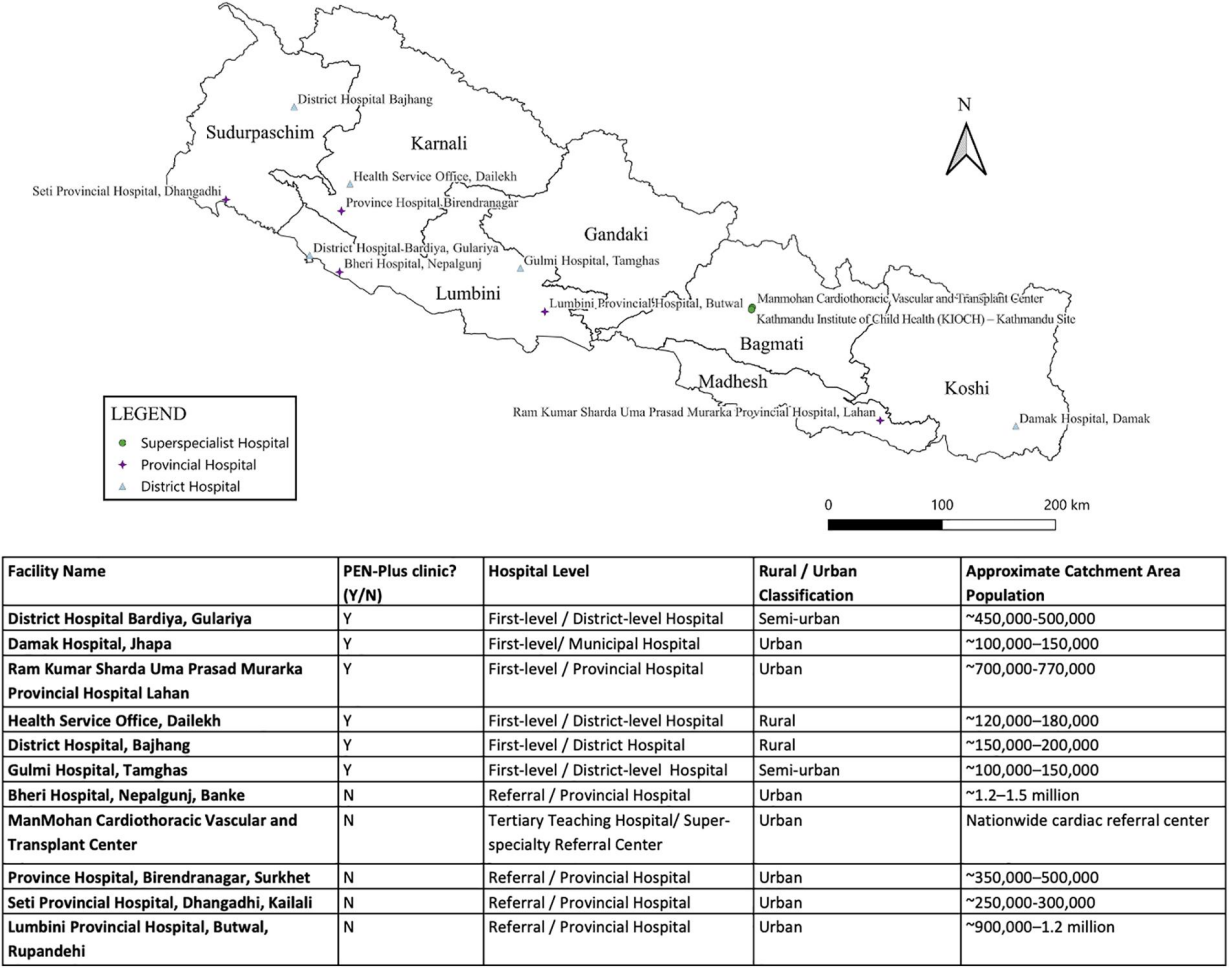
Location of participating facilities in Nepal and characteristics of each facility.

The 11 participating facilities are located across Nepal as pictured in Figure 2, and characteristics of each facility are listed. Together, the participating district and provincial hospitals serve an estimated combined catchment population of approximately 4–5 million people across urban, semi-urban, and rural regions of Nepal, or 1/6 of Nepal's total population.

### 2.5 Study population

The target population includes children and adolescents aged 5–18 years presenting to participating facilities or found in community outreach with fever, joint pain, or other symptoms suggestive of ARF which would warrant full screening using the Jones criteria (13), as well as a history of sore throat. Children presenting at the listed facilities will be screened for ARF and RHD. Children diagnosed in schools or other community settings will be referred to the nearest participating facility for evaluation.

### 2.6 Intervention description and procedures

#### 2.6.1 Community case-finding procedures

To address poor health-seeking behavior and limited community awareness of ARF, a community-based case-finding approach will be implemented across participating districts. Female Community Health Volunteers (FCHVs), supported by trained healthcare workers, will conduct community-level awareness activities focusing on sore throat, early ARF symptoms (predominantly a combination of fever and joint pain), potential complications, and the importance of timely treatment. Following sensitization, FCHVs will undertake symptom-based screening and basic clinical assessments during routine community visits.

Referral to a participating facility is triggered by the presence of symptom combinations consistent with possible ARF, primarily fever with joint involvement, rather than any single symptom in isolation; a standardized symptom checklist was distributed to FCHVs and school personnel during training to guide this assessment.

In parallel, a school-based approach will complement community-level case detection. School nurses and teachers will be oriented on ARF symptom identification, early diagnosis and treatment, and referral pathways. Following sensitization, trained healthcare workers will conduct clinical screening, including cardiac auscultation and abbreviated echocardiography protocols using portable machines, in schools within participating districts.

#### 2.6.2 Clinical evaluation workflow

ARF cases will be diagnosed if children meet 2015 modified Jones criteria (13) for high-risk populations. Nurses and physicians at participating facilities will confirm the diagnosis through laboratory tests, physical examination, and echocardiography. ARF diagnostic criteria included in SOPs and REDCap registry forms guide providers in confirming diagnostic criteria for enrollment.

Cases that are determined to be likely ARF are entered into the registry and their echocardiograms are uploaded for review by cardiologists. There are several participating cardiologists at tertiary care facilities in the capital city of Kathmandu who will provide diagnostic support and clinical advice as requested by study clinicians. Recruitment and follow-up will occur continuously, with structured follow-up visits scheduled at 3, 6, and 12 months post-enrollment for each ARF patient. Children requiring penicillin prophylaxis will be reviewed clinically in accordance with national follow-up guidelines. Follow-up will occur at the closest participating facility to the child's residence.

At each participating site, implementation is led by a designated clinical team comprising a physician, staff nurse, and laboratory technician. This team is responsible for application of ARF diagnostic criteria, echocardiographic assessment, registry documentation, and coordination of follow-up care.

Training was held by KIOCH in November 2025 during which SOPs for recruitment, ARF diagnostic criteria, and echocardiography protocols were reviewed with all providers and distributed. All facilities are expected to adhere to centrally defined SOPs and any deviations or contextual constraints will be documented in detail and reviewed during monthly monitoring and quality assurance meetings with the central KIOCH team.

#### 2.6.3 ARF diagnostic criteria

The 2015 modified Jones criteria (13) for high-risk populations will be used. The diagnosis of ARF is made if two major or one major and two minor criteria are met along with the evidence of streptococcal infection. If ARF is thought to be a recurrent episode, three minor criteria can be used to make the diagnosis. Major criteria include presence of carditis, mono- or polyarthritis, polyarthralgia, chorea, erythema marginatum, and subcutaneous nodules. Minor criteria include monoarthralgia, fever ≥38.0 °C, elevated inflammatory markers: ESR ≥30 mm/h and/or CRP ≥3.0 mg/dL, or prolonged PR interval on ECG. Prolonged PR interval is counted as a minor criterion only if carditis is not being used as a major criterion. There must be evidence of preceding group A streptococcal infection which is required for all except isolated chorea or indolent carditis. Antistreptolysin O Titer (ASOT) testing is provided to all facilities by the PRIMA study and will be the most common GAS antibody test used, though anti-DNase B titer, positive throat culture for GAS, and positive rapid antigen detection testing for GAS can also be used.

Carditis may be clinical or subclinical. Subclinical carditis is defined by Doppler echocardiographic evidence of pathological mitral or aortic regurgitation and morphologic changes as detailed in the 2023 World Heart Federation guidelines for the echocardiographic diagnosis of RHD (14). Echocardiographic assessments are performed using standardized imaging checklists which specifically include the criteria for pathological regurgitation and morphologic change. Echocardiograms are all uploaded for central review by cardiologists to support diagnostic consistency and fidelity. Kathmandu-based cardiologists serve as the final interpretive authority for echocardiographic findings, consistent with specialist referral practice within Nepal's health system. All clinics are equipped with a cardiac ultrasound machine.

Cases in which diagnostic criteria are not clearly met are reviewed by a study cardiologist, and children may be classified as suspected or probable ARF when appropriate. Diagnostic classification may be updated over time as additional clinical, laboratory, or echocardiographic information becomes available during follow-up, which reflects real-world practice.

Procedures related to recruitment of controls and biomarker analysis (blood sample collection) as part of the larger multi-national PRIMA study will be collected, however they are not the focus of this implementation analysis and thus are not described.

### 2.7 Data collection and management

Clinical data from initial consultation will be collected using standardized electronic case report forms in REDCap, which comprises the registry. Echocardiographic images will be exported from the ultrasound machine to Tricefy, an online cloud-based repository, and linked by unique study IDs. Patients who have positive echocardiograms will be followed up at their local health facility monthly per clinic protocols, and at 3, 6, and 12 months they will be followed up per study protocols using a REDCap form. Echocardiographic quality will be monitored by cardiologists, and constructive feedback will be provided to performing physicians at the recruiting facilities as needed. Program delivery and data quality will be monitored through periodic review of REDCap registry completeness and monthly quality check-ins will be conducted by the central KIOCH team.

### 2.8 Outcome measures and data sources

Evaluation of the ARF case-finding program will use Proctor's taxonomy of implementation outcomes (8) with a primary focus on acceptability, feasibility, and adoption during early program delivery across diverse health system contexts in Nepal. Implementation outcomes are summarized in Table 1. Definitions and examples of Proctor's taxonomy outcomes are provided in Supplementary Table 1. These outcomes will be assessed primarily at the facility level to reflect integration of the standardized workflow into routine clinical practice. Implementation outcomes will be evaluated from April 2026 to April 2027, following a two-month run-in period.

**Table 1:** Implementation outcomes evaluated in this study protocol, organized according to Proctor's taxonomy (8) with corresponding units of analysis, key evaluation questions, data elements, and data sources. The final row describes exploratory care-process indicators assessed at the patient and facility level during the first 12 months of program delivery; these are not Proctor outcomes and are not intended to evaluate clinical effectiveness. ARF, acute rheumatic fever; FCHV, Female Community Health Volunteer; QA, quality assurance; REDCap, Research Electronic Data Capture; SOP, standard operating procedure.

Proctor outcome(s)	Unit of analysis	Key questions addressed	Data elements	Data sources
Penetration	Facility/catchment	Are children with symptoms suggestive of ARF being identified and evaluated within routine care?	Number of children evaluated for ARF per site Number and characteristics of children enrolled with suspected or confirmed ARF Distribution by age, sex, distance to facility, and referral source	REDCap clinical data Facility clinical records
Acceptability	Provider/facility	Is the program acceptable to the people delivering it? Do providers and hospital administrators think it is adding to their workload.	Do providers agree with the program? Do providers think it is adding to the workload? Provider-reported perceived usefulness and acceptability	Provider/FCHV structured surveys Hospital superintendent structured surveys
Adoption	Facility/provider	Have participating facilities initiated and operationalized the standardized ARF case-finding and registry workflow?	Number and proportion of facilities initiating the full workflow Number and cadre of providers participating	Facility clinical records Provider structured surveys
Feasibility	Facility/provider	Can the standardized workflow be delivered within routine inpatient and outpatient care?	Reported workflow barriers Time burden estimates Documentation of contextual constraints	Monthly monitoring reports Provider structured surveys
Fidelity	Facility/provider	Are ARF diagnostic and registry procedures implemented according to centrally defined SOPs?	Proportion of evaluations meeting Jones criteria documentation standards Echocardiography completeness and image adequacy Registry completeness Frequency and nature of protocol deviations	REDCap clinical data Monthly monitoring reports
Sustainability	Facility	Is the program still operational with sustained adherence at 12 months?	Continued ARF case enrollment activity Continued registry use Ongoing participation in QA processes Sustained workflow adherence	REDCap clinical data Monthly monitoring reports
—	Patient/Facility	How is the standardized workflow functioning clinically during early implementation?	Number and proportion of ARF diagnoses among evaluated children Diagnostic concordance with central cardiologist review Time from symptom onset to diagnosis Time from diagnosis to initiation of prophylaxis Follow-up completion at 3, 6, and 12 months	REDCap clinical data Facility clinical records

Exploratory care-process indicators will be assessed at the patient level and are intended to characterize whether the intervention may function to improve ARF care in the absence of pre-implementation comparator data. These include the number and proportion of ARF diagnoses among evaluated children, accuracy and timeliness of diagnosis, linkage to follow-up care, and follow-up completion over time. These measures are descriptive and are not intended to evaluate long-term clinical effectiveness.

### 2.9 CFIR determinants

Although the primary focus of this study is evaluation of acceptability, feasibility, and adoption, selected domains of the Consolidated Framework for Implementation Research (CFIR) will be used to structure interpretation of implementation experience across recruiting facilities (Table 2). Given the standardized nature of the program and the emphasis on adherence to centrally defined SOPs, CFIR is applied in a focused manner to identify contextual factors that may influence fidelity and maintenance. Determinant data will be derived from routine program monitoring, structured provider surveys, notes from monthly quality assurance calls, and documentation of protocol deviations. This approach allows systematic interpretation of barriers and facilitators without introducing intensive qualitative data collection or additional burden to participating facilities focused on program implementation. CFIR constructs are therefore applied deductively as an interpretive lens rather than as the basis for inductive qualitative analysis; free-text survey responses will be summarized descriptively.

**Table 2:** Selected CFIR domains used to interpret implementation feasibility and fidelity.

CFIR domain	Constructs	Data sources	Role in analysis
Intervention characteristics	Perceived complexity of ARF criteria; clarity of SOPs; registry usability	Provider structured surveys; frequency of protocol deviations; registry completeness	Explain fidelity variation
Inner setting	Leadership engagement; staffing capacity; competing clinical priorities	Monthly monitoring calls; structured surveys; site logs; documentation of workflow barriers	Interpret feasibility differences
Outer setting	Referral networks; geographic barriers	Referral source data; catchment descriptions; distance to facility	Contextualize reach
Characteristics of individuals	Provider knowledge; confidence in ARF diagnosis; perceived value of program	Provider surveys	Assess adoption, acceptability and sustained engagement
Implementation process	Training adequacy; quality assurance feedback; monitoring intensity	Cardiologist echo over-read reporting form; structured surveys; notes from monthly reviews	Examine mechanisms supporting fidelity

#### 2.9.1 Data sources

Data sources include facility clinical records, REDCap clinical data, provider and hospital superintendent surveys, and monthly monitoring reports with site-based study leads.

Facility clinical records: We will examine the routine paper-based clinical forms that exist in the facilities. We will examine background data on participants, as well as monthly records for participants who are positive for ARF. At the end of the program, researchers will hand search clinical forms to extract relevant data.

REDCap clinical data: Three types of REDCap clinical data will be used for this study.

1. When a suspected case is referred to a participating facility, the study encounter and suspected diagnosis will be recorded in REDCap.
2. If the case screens positive for ARF, their echocardiogram will be reviewed by a Kathmandu-based cardiologist who will report their echocardiographic findings in REDCap.
3. Per study protocol, at three study points (3, 6, and 12 months) patients diagnosed with ARF will be monitored for progress. These visits will be conducted using REDCap forms.

Provider surveys: Surveys will be distributed at 12 months to all providers (one doctor and one nurse at each facility). Surveys will consist of a mix of multiple choice, Likert-scale and free text questions. These will be conducted in a mix of English and Nepali and will be conducted through Google forms.

Hospital superintendent surveys: Surveys will be distributed at 12 months to the superintendent in charge of each facility enrolled in this study. Surveys will consist of a mix of multiple choice, Likert-scale and free text questions. These will be conducted in a mix of English and Nepali and will be conducted through Google forms.

Monthly monitoring reports: Once a month, a central researcher from KIOCH will hold a “monthly check-in” with each of the 11 site-based study leads. These free-form discussions will cover barriers and solutions encountered over the past month, as well as number of cases, perceptions of case mix, and any questions that have arisen. These check-ins will not have audio recording but the KIOCH researcher will take detailed notes.

### 2.10 Analysis

Analyses will be descriptive and exploratory, consistent with the pragmatic, non-randomized design and primary focus on implementation outcomes. Acceptability, adoption, feasibility, fidelity, penetration, and short-term sustainability will be summarized at the facility level using appropriate summary statistics. Results will be reported individually by facility and stratified by facility type (district vs. referral hospital) to characterize variation in implementation experience across site types.

Acceptability will be assessed through a simple summary statistics including means, and ranges of scores from providers surveys, and through a quantifying data found in provider and hospital superintendent surveys.

Adoption will be assessed through a narrative description of how workflows were deployed across facilities using data from provider surveys, REDCap clinical records, and facility clinical records.

Feasibility will be assessed quantitively through the percentage of workflows that are completed using data from REDCap clinical records and qualitatively through barriers reported in the provider structured surveys and monthly monitoring reports.

Fidelity will be quantified using adherence metrics including completeness of RedCap registry forms, echocardiographic protocol compliance, and documented protocol deviations from monthly monitoring reports.

Short-term sustainability will be evaluated quantitatively based on continued case enrollment, REDCap registry use, and participation in quality assurance processes at 12 months using data from REDCap activity logs and monthly reports.

Exploratory care-process indicators will be summarized at the patient level, including diagnostic patterns, concordance with central review, timeliness of diagnosis, and follow-up completion. In cases where initial diagnosis is unclear and reclassification occurs, final diagnoses will be used where possible. These measures are descriptive and not intended to assess clinical effectiveness. The study is not designed for formal between-facility comparisons. Patient-level registry data, provider-level survey data, and facility-level monitoring reports serve complementary descriptive roles and are not formally integrated through multilevel modeling, consistent with the pragmatic, single-arm design.

As this is an early-phase descriptive study, no *a priori* thresholds for successful implementation are defined; outcomes will be interpreted descriptively and in light of contextual factors identified through CFIR domains.

### 2.11 Ethical considerations

The study was approved on December 1, 2025, by the Nepal Health Research Council. Written informed consent will be obtained from parents or guardians, and assent from children aged 12 years or older for permission to participate in the PRIMA research study and for registry enrollment. Parents and children who do not wish to participate in the study will continue to receive routine care as medically indicated without inclusion in the registry.

## 3 Discussion

This protocol describes the implementation of a standardized ARF case-finding and registry program embedded within routine district- and referral-level care across 11 facilities in Nepal. The primary aim is to evaluate early implementation outcomes and explore contextual factors influencing implementation. In a setting where ARF is frequently under-recognized and follow-up fragmented, establishing a consistent operational workflow represents a necessary first step toward earlier detection.

Despite a high prevalence of RHD, Nepal lacks a coordinated system for early identification of suspected ARF and registry-based tracking of follow-up and secondary prophylaxis. By integrating ARF evaluation into district-level services aligned with PEN-Plus infrastructure, this program aims to strengthen routine clinical capacity rather than introduce stand-alone screening programs that may be difficult to sustain. Earlier identification of ARF may enable timely initiation of secondary prophylaxis, which reduces progression to valvular heart disease. The emphasis on centralized training, clearly defined SOPs, and ongoing quality assurance reflects a deliberate focus on fidelity and reproducibility during early implementation. Over time, this infrastructure may also enable more consistent monitoring of disease progression and outcomes and, if successful, may inform future national scale-up.

Several challenges are anticipated. Diagnosis of acute rheumatic fever remains clinically complex, particularly in high-burden settings where presentations may be heterogeneous and overlapping infectious etiologies are common. Although the 2015 modified Jones criteria provide standardized guidance, diagnosis relies on combinations of clinical findings, laboratory evidence, and echocardiographic interpretation that may be incomplete or evolving at initial presentation. Consequently, some children may initially be classified as indeterminate or probable ARF, with diagnostic status evolving as additional clinical information becomes available. Staff turnover, variation in infrastructure, and high patient volume across participating facilities may further influence adoption and fidelity of the standardized workflow. Geographic barriers and socioeconomic constraints may affect retention in follow-up independent of program performance. For these reasons, this study prioritizes documentation of contextual factors and structured assessment of workflow adherence during the first 12 months of implementation.

Additionally, the symptom-triggered case-finding approach used in this program will not identify children with subclinical carditis detectable only by echocardiography in the absence of clinical symptoms. Population-based echocardiographic screening would be required to capture this group; however, such programs are resource-intensive and not feasible for routine implementation within most health systems in low-resource settings. The purpose of this program—and of this implementation study—is to evaluate whether structured ARF case-finding and follow-up can be pragmatically embedded within existing district-level health system infrastructure, rather than to conduct population-level RHD surveillance.

Although this protocol does not evaluate long-term clinical effectiveness, the implementation data generated will inform future analyses examining diagnostic patterns, barriers and facilitators to program delivery, and additional Proctor outcomes such as cost. If feasible and sustainable within the Nepalese health system, this approach may offer a pragmatic model for other RHD-endemic low- and middle-income countries seeking to embed ARF detection within decentralized care platforms where high disease burden, limited specialist availability, and fragmented follow-up remain common challenges.

## Data Availability

The original contributions presented in the study are included in the article/Supplementary Material, further inquiries can be directed to the corresponding authors.
