# Supplementary material for: Implementing a national acute rheumatic fever case-finding program and registry in Nepal: protocol for a multi-site implementation study
Source: Front Cardiovasc Med. 2026 May 7;13:1823957. doi: 10.3389/fcvm.2026.1823957 (PMC13190454; doi:10.3389/fcvm.2026.1823957)
Supplement: Supplementary file 1 [file Table1.docx]

| **Outcome** | **Definition** | **Example in this study** |
| --- | --- | --- |
| Acceptability | Perceived appropriateness of the program among providers and facility leadership | Whether nurses and physicians at district hospitals find the ARF case-finding workflow compatible with existing clinical routines |
| Adoption | Initiation and active use of the standardized workflow across participating facilities | Whether all 11 facilities have begun enrolling suspected ARF cases into the REDCap registry within the study period |
| Feasibility | Extent to which the workflow can be successfully carried out within existing clinical systems | Whether echocardiography, laboratory testing, and registry documentation can be completed for suspected ARF cases within routine outpatient or inpatient visits |
| Fidelity | Degree of adherence to ARF diagnostic criteria, echocardiographic protocols, and registry documentation requirements | Completeness of REDCap registry forms and concordance with Jones criteria documentation across participating facilities |
| Penetration | Reach of the program within the catchment populations of participating facilities | Number of suspected ARF cases identified relative to estimated facility catchment population across urban, semi-urban, and rural sites |
| Short-term sustainability | Continued program operation and workflow adherence at 12 months | Whether facilities maintain active case enrollment and participation in monthly quality assurance calls through April 2027 |

Supplemental Table S1. Definitions and study-specific examples of implementation outcomes based on Proctor's taxonomy. ARF, acute rheumatic fever; REDCap, Research Electronic Data Capture. Definitions adapted from Proctor E et al., Adm Policy Ment Health 2011. (8)
